# Methanolic Extracts of *Solieria* *robusta* Inhibits Proliferation of Oral Cancer Ca9-22 Cells via Apoptosis and Oxidative Stress

**DOI:** 10.3390/molecules191118721

**Published:** 2014-11-14

**Authors:** Yii-Huei Yen, Ammad Ahmad Farooqi, Kun-Tzu Li, Ghazala Butt, Jen-Yang Tang, Chang-Yi Wu, Yuan-Bin Cheng, Ming-Feng Hou, Hsueh-Wei Chang

**Affiliations:** 1 Department of Dentistry, Ten Chan General Hospital, Chung-Li 32043, Taiwan; E-Mail: theericyen@yahoo.com; 2 Laboratory for Translational Oncology and Personalized Medicine, Rashid Latif Medical College, Lahore 54000, Pakistan; E-Mail: ammadfarooqi@rlmclahore.com; 3 Department of Biomedical Science and Environmental Biology, Kaohsiung Medical University, Kaohsiung 80708, Taiwan; E-Mail: sherry30126@yahoo.com.tw; 4 Department of Botany, Government College University, Lahore, Katchery Road Lahore 54000, Pakistan; E-Mail: drghazala71@yahoo.com; 5 Cancer Center, Kaohsiung Medical University Hospital, Kaohsiung Medical University, Kaohsiung 80708, Taiwan; E-Mail: reyata@kmu.edu.tw; 6 Department of Radiation Oncology, Faculty of Medicine, College of Medicine, Kaohsiung Medical University, Kaohsiung 80708, Taiwan; 7 Department of Radiation Oncology, Kaohsiung Medical University Hospital, Kaohsiung 80708, Taiwan; 8 Department of Radiation Oncology, Kaohsiung Municipal Ta-Tung Hospital, Kaohsiung 80145, Taiwan; 9 Department of Biological Sciences, National Sun Yat-Sen University, Kaohsiung 80424, Taiwan; E-Mail: cywu@mail.nsysu.edu.tw; 10 Graduate Institute of Natural Products, College of Pharmacy, Kaohsiung Medical University, Kaohsiung 80708, Taiwan; E-Mail: jmb@kmu.edu.tw; 11 Institute of Clinical Medicine, Kaohsiung Medical University, Kaohsiung 80708, Taiwan; 12 Department of Surgery, Kaohsiung Municipal Ta-Tung Hospital, Kaohsiung 80145, Taiwan; 13 Institute of Medical Science and Technology, National Sun Yat-sen University, Kaohsiung 80424, Taiwan; 14 Research Center of Excellence for Environmental Medicine, Kaohsiung Medical University, Kaohsiung 80708, Taiwan

**Keywords:** red alga, oral cancer, apoptosis, ROS, mitochondrial depolarization

## Abstract

Many red algae-derived natural products are known to have anticancer effects. The biological functions of the red alga *Solieria* *robusta* from the Karachi coast (Pakistan) remain unclear. Here, we prepared a methanolic extracts of *S. robusta* (MESR) to examine its possible anti-oral cancer effects and the corresponding mechanism of action. Cell viability of MESR-incubated oral cancer Ca9-22 cells was dose-responsively decreased (*p* < 0.001). According to a propidium iodide (PI)-based assay the cell cycle distribution was dramatically changed, especially for subG1 accumulation. Annexin V/PI assay of apoptosis using flow cytometry also showed that MESR-incubated Ca9-22 cells were dose-responsively increased (*p* < 0.001). For evaluation of oxidative stress in MESR-incubated Ca9-22 cells, we found that reactive oxygen species (ROS) were overexpressed dose- and time-responsively and mitochondrial depolarization was also increased (*p* < 0.001). Taken together, MESR showed inhibitory effects on oral cancer proliferation coupled with apoptosis and oxidative stress.

## 1. Introduction

The occurrence of oral cancers is high in Asian countries, including Taiwan. Although some tumor markers [1,2] of oral cancer have been reported for potential therapeutic targets, the main strategy for oral cancer therapy is still drug treatment. Recently, the strategy of combined treatment for cancer treatment is favored [3,4], making the identification of more potential drugs or natural products as candidates in drug combinations helpful. Mounting evidence shows that marine algae extracts and components have anti-cancer effects [5], for example green [6], brown [7], blue-green [8], and red algae [9]. Lycopene isolated from the green alga *Chlorella marina* displayed considerable anticancer activity against PC-3 and DU-145 prostate cancer cell lines [10]. The methanolic extracts of a brown alga *Sargassum* *muticum* reportedly inhibited the MCF-7 and MDA-MB-231 breast cancer cell lines [11]. A blue-green alga *Spirulina* *platensis* is a source of tetrapyrrolic components with notable activity against tumor growth in mice xenografted with pancreatic cancer cells [12]. Aqueous extracts of a red alga *Gracilaria* *corticata* [10] displayed the antiproliferative effect against human leukemic cells.

We have focused on the biological functions of red algae. Previously, we found that both ethanolic [13] and methanolic [14] extracts of the red alga *Gracilaria* *tenuistipitata* caused growth inhibition of oral cancer cells. Similarly, a methanolic extracts of *Plocamium* *telfairiae* displayed antiproliferative effects against colon cancer cells [15]. The red alga *Agardhiella* *robusta*, officially named *Solieria* *robusta*, can effectively reduce the lipid profile of diet-induced hyperlipidaemic rats [16]. According to the NCBI taxonomy browser [17], the species *S.* *robusta*, belonging to the family Solieriaceae contains 15 genera of red algae. Among them, *S.* *robusta*, *Eucheuma* *serra*, and *Kappaphycus* *alvarezii* are the main industrial resources of the polysaccharide carrageenan [18], a common food industry and medicinal supplement [19]. However, the possible anticancer function of the red alga *S.* *robusta* remains unclear. Because *S.* *robusta* is abundant along the Karachi coast of Pakistan, it is convenient to prepare methanolic extracts of *S.* *robusta* (namely for MESR). In this study, we aimed to explore the biological function of MESR toward oral cancer cells by analyzing their cell proliferation, cell cycle changes, apoptosis, and oxidative stress.

## 2. Results

### 2.1. Cell Viability of MESR-Incubated Ca9-22 Cells

**Figure 1:**
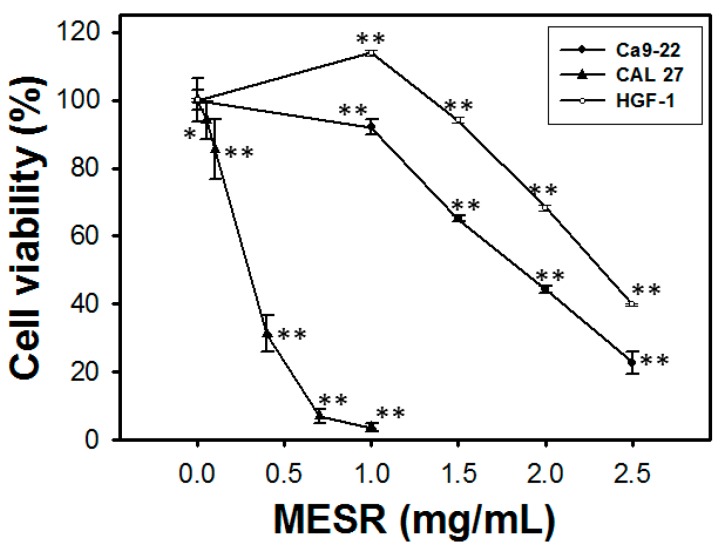
MTS-based cell viability of oral cancer Ca9-22 and CAL 27 cells as well as oral normal HGF-1 cells was differentially decreased by MESR. Cells were incubated with MESR (0–2.5 mg/mL) for 24 h. Data, means ± SDs (*n* = 11, 10, and 3, respectively). *p < 0.001 and ** *p* < 0.001 against control.

In oral cancer Ca9-22 (Figure 1), the relative cell viabilities (%) in terms of MTS assay for MESR treatments (0, 1, 1.5, 2, and 2.5 mg/mL) after 24 h were 100.0 ± 2.9, 92.1 ± 2.3, 65.2 ± 1.0, 44.3 ± 1.1, and 22.8 ± 3.2, respectively. In oral cancer CAL 27 cells, the relative cell viabilities (%) in terms of MTS assay for MESR treatments (0, 0.05, 0.1, 0.4, 0.7, and 1 mg/mL) after 24 h were 100.0 ± 6.3, 94.1 ± 5.6, 85.5 ± 8.8, 31.3 ± 5.3, 7.0 ± 1.9, and 3.7 ± 1.3, respectively. In oral normal HGF-1 cells, the relative cell viabilities (%) in terms of MTS assay for MESR treatments (0, 1, 1.5, 2, and 2.5 mg/mL) after 24 h were 100.0 ± 0.5, 113.9 ± 0.8, 94.1 ± 0.8, 68.4 ± 0.9, and 39.9 ± 0.3, respectively. Accordingly, cell viabilities of MESR-incubated oral cancer Ca9-22 and CAL 27 cells were dose-responsively decreased and treatment was less harmful to normal oral HGF-1 cells (*p* < 0.05–0.001 compared to control).

### 2.2. Cell Cycle Distribution of MESR-Incubated Ca9-22 Cells

**Figure 2:**
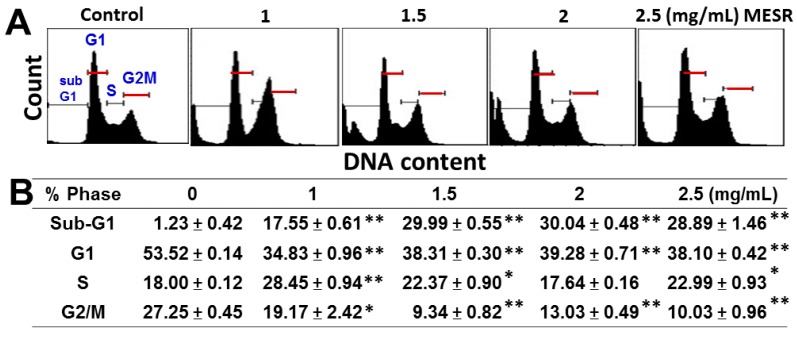
Changes of the cell cycle distribution of MESR-incubated oral cancer Ca9-22 cells. Cells were incubated with MESR (0–2.5 mg/mL) for 24 h for flow cytometry analysis. (**A**) Typical cell cycle patterns of MESR-incubated Ca9-22 cells. (**B**) Statistics of cell cycle phases (%) for Figure 2A. Data, means ± SDs (*n* = 3). * *p* < 0.05; ** *p* < 0.001 against control.

The cell cycle patterns of flow cytometry of MESR-incubated cells are shown in Figure 2A. After MESR treatment (Figure 2B), the sub-G1 populations were dose-responsively increased in MESR-incubated oral cancer Ca9-22 cells (*p* < 0.001). G1 and G2/M phases were dramatically decreased and S phase was slightly increased after MESR treatments (*p* < 0.05–0.001).

### 2.3. Apoptosis Analysis of MESR-Incubated Ca9-22 Cells

**Figure 3:**
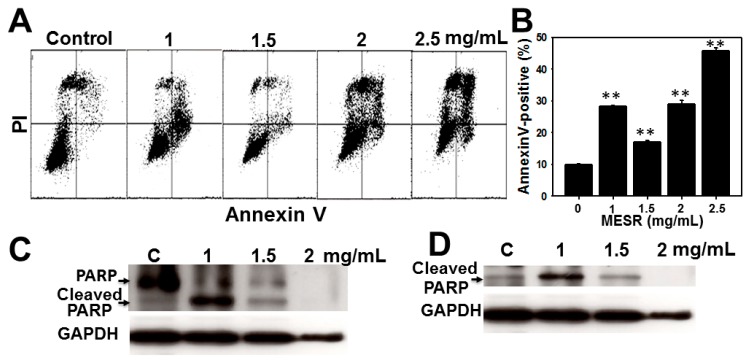
Changes of apoptosis of MESR-incubated oral cancer Ca9-22 cells. Cells were incubated with MESR (0–2.5 mg/mL) of for 24 h for flow cytometry and western blotting analyses. (**A**) Typical patterns of annexin V/PI method for MESR-incubated Ca9-22 cells. (**B**) Apoptosis statistics (%) in Figure 3A. Data, means ± SDs (*n* = 3). ** *p* < 0.001 against control. (**C** and **D**) western blotting of the uncut PARR and the apoptotic marker of cleaved PARP in MESR-treated Ca9-22 cells, respectively.

The flow cytometry annexin V/PI patterns of MESR-incubated cells are displayed in Figure 3A. In Figure 3B, the annexin V-positive expression (%) for MESR treatment of oral cancer Ca9-22 cells was dose-responsively increased (*p* < 0.001). 

For western blotting, the expressions of uncut poly ADP-ribose polymerase (PARP) were decreased and the apoptotic marker of the cleaved PARP [20] were increased by MESR treatment at 1 and 1.5 mg/mL compared to control (Figure 3C,D), respectively.

### 2.4. ROS Changes of MESR-Incubated Ca9-22 Cells

**Figure 4:**
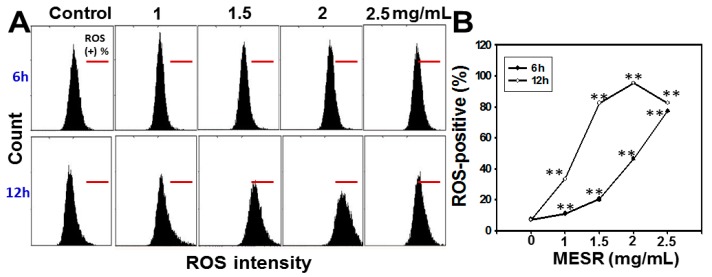
Changes of ROS levels of MESR-incubated oral cancer Ca9-22 cells. Cells were incubated with MESR (0–2.5 mg/mL) for 6 h and 12 h for flow cytometry analysis. The horizontal red lines in each plot indicated ROS-positive (%). (**A**) Typical ROS patterns of MESR-incubated Ca9-22 cells. (**B**) Statistics of ROS-positive intensity (%) in Figure 4A. Data, means ± SDs (*n* = 3). ** *p* < 0.001 against control.

The ROS staining-positive patterns of MESR-incubated Ca9-22 cells are displayed in Figure 4A. After MESR treatments for 6 h and 12 h, the ROS staining-positive expression (%) of MESR-incubated oral cancer Ca9-22 cells was significantly accumulated in both dose- and time-responsive manners (*p* < 0.001) (Figure 4B).

**Figure 5:**
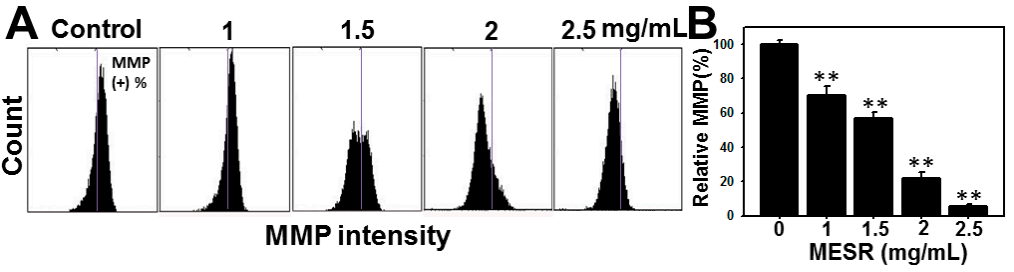
Changes of MMP levels of MESR-incubated Ca9-22 oral cancer cells. Cells were incubated with MESR (0–2.5 mg/mL) for 24 h for flow cytometry analysis. The vertical lines in each plot dichotomised the MMP intensity scales into 50%/50% in reference to the left and right sides of control. The right side of MMP intensity was regarded as MMP-positive (%). (**A**) Typical MMP patterns of MESR-incubated Ca9-22 cells. (**B**) Statistics of relative MMP-positive intensity (%) in Figure 5A. All the MMP intensity data were normalized to control as 100% for the relative MMP (%) calculation. Data, means ± SDs (*n* = 3). ** *p* < 0.001 against control.

### 2.5. MMP Changes of MESR-Incubated Ca9-22 Cells

The MMP positive patterns of flow cytometer of MESR-incubated cells are displayed in Figure 5A. After MESR for 24 h, the MMP-positive expression (%) of oral cancer Ca9-22 cells was dose-responsively decreased (*p* < 0.001) (Figure 5B).

### 2.6. Discussion

Most marine algae are the common antioxidant-rich edible plants, especially their organic solvent extracts [21,22]. Although antioxidants were reported to have many benefits for chemoprevention [23,24], accumulating evidence shows that antioxidants may induce DNA damages, mutagenicity, and cell death [25,26]. Consistent with our results, the antiproliferative effect of MESR against oral cancer Ca9-22 cells was firstly demonstrated.

Many studies of methanol extracts of red algae also show the growth inhibitory effects against cancers. For example, the IC_50_ value of methanol extracts of red alga *Plocamium* *telfairiae-*treated colon cancer HT-29 cells after 24 h incubation was 2 µg/mL [15]. Methanol extracts of the red alga *Halurus* *equisetifolius* exhibited the IC_50_ values of 0.075, 0.060 and 0.175 mg/mL for human lung A549, colon HCT15 and breast MCF7 cancer cell lines, respectively [27]. The IC_50_ value of methanol [14] extracts of the red alga *Gracilaria* *tenuistipitata*-treated Ca9-22 cells after 24 h treatment was 0.326 mg/mL, respectively. In the present study, the IC_50_ values of the MESR-incubated Ca9-22 cells after 24 h were 1.89 mg/mL. For other oral cancer cell line CAL 27, its IC_50_ value was 0.296 mg/mL after 24 h treatment. Therefore, the dosages for growth inhibitory effects may differ for different species of red algae and cancer types. Although MESR showed higher a IC_50_ value than other red algae methanol extracts as previously described, it may be helpful for cancer treatment considering the possible synergy effects. Combination of different natural product extracts and bioactive constituents may generate synergistic multi-target effects [28,29].

Moreover, the IC_50_ value of MESR in normal oral HGF-1 cells is 2.32 mg/mL and of Ca9-22 cells is 1.89 mg/m, therefore MESR is more cytotoxic to Ca9-22 cells but less harmful to normal HGF-1 cells, especially for the concentrations ranging from 0 to 1.5 mg/mL. Under the IC_50_ value of MESR of Ca9-22 cells, the normal HGF-1 cells are still 80% viable. Therefore, the MESR has the selective killing potential for oral cancer therapy at suitable concentrations.

ROS induction is known as one of the causes for cancer cell death [30,31]. Induction of oxidative stress from drugs such as cisplatin and doxorubicin may have genotoxicity for cancer therapy [26,32]. Extracts of red alga *Jania* *longifurca* displayed significantly higher anticancer activity against MCF-7 breast cancer cells, primarily by enhancing oxidative stress and apoptosis in cancer cells [33]. During apoptosis, it was reported that discontinuous fragmentation of nuclear DNA may display discrete sub-G1 peaks using PI staining-based flow cytometer analysis [34]. Likewise, we found that MESR can induce subG1 accumulation and apoptosis in oral cancer Ca9-22 cells using flow cytometry analysis and western blotting. Based on the morphological hallmarks of apoptosis, using a microscope we also found that the MESR-induced cell death of OSCC Ca9-22 displays physiological biomarkers of apoptosis, including membrane blubbing, cell rounding and the formation of apoptotic bodies in a dose-response manner (data not shown). Moreover, MESR can induce ROS overproduction and mitochondrial dysfunction in OSCC Ca9-22. Therefore, oxidative stress may play a role in growth inhibition of oral cancer Ca9-22 cells after MESR treatment.

## 3. Experimental Section 

### 3.1. Cell Cultures and Methanolic Extracts of S. robusta 

A human oral gingival cancer cell line (Ca9-22), purchased from the Health Science Research Resources Bank (Osaka, Japan), was maintained in DMEM/F12 (3:2) medium (Gibco, Grand Island, NY, USA) containing 10% fetal bovine serum (Gibco), penicillin/streptomycin, and 0.03% glutamine under 37 °C with humidified 5% CO_2_.

*S. robusta* was collected from the Karachi coast, Pakistan. The species was identified from available literature and the specimen was confirmed by its Herbarium Sheet from Karachi University and authenticated by the phycologist Dr. Mustafa Shameel. After carefully washing in running water to avoid marine contaminants, the red alga were dried, ground, and stored in airtight containers before use. Powdered shade-dried red algal samples (500 g of *S. robusta*) were extracted at room temperature with methanol (1500 mL) for three weeks and then they were filtered through Whatman filter paper (nos. 1, 2, 41, and 42) to collect clarified filtrates (1 L). Finally, they was evaporated (65–70 °C) under vacuum to produce a dark green viscous oily mass (17.34 g) of methanolic extract of *S.* *robusta* (MESR). The NMR spectrum revealed signals in the *δ*_H_ 3.0–4.0 region representing the sugar moiety and signals in the *δ*_H_ 0.5–2.5 region representing fatty acids (data not shown). It was dissolved in 0.1% dimethyl sulfoxide (DMSO) before treatment.

### 3.2. Cell Viability 

Cell viability was detected by a CellTiter 96^®^ AQueous One Solution Cell Proliferation Assay (MTS, Promega Corporation, Madison, WI, USA) as described [35]. In brief, cells after seeding overnight were incubated with MESR (0, 1, 1.5, 2 and 2.5 mg/mL) or DMSO (0.025%) as a control for 24 h. Subsequently, the MTS assay was performed and results recorded by an ELISA plate reader.

### 3.3. Cell Cycle Distribution 

Cell cycle analysis was based on DNA staining method as described [36]. In brief, cells were incubated with0, 1, 1.5, 2 and 2.5 mg/mL of MESR for 24 h. After harvest and PBS washing, cells were fixed with 70% ethanol and harvested by centrifugation. Subsequently, cells were resuspended in PBS solution containing 50 µg/mL of propidium iodide (PI, Sigma, St Louis, MO, USA) and waited for 30 min at 37 °C in darkness. Cell cycle phases were determined by a FACS Calibur flow cytometer (Becton-Dickinson, Mansfield, MA, USA) and FlowJo software (version 10) (Tree Star Inc., Ashland, OR, USA).

### 3.4. Apoptosis Analysis

Apoptosis was monitored by annexin V (Strong Biotect Corporation, Taipei, Taiwan)/PI (Sigma) method as described [37,38]. In brief, cells after seeding overnight were treated with or without MESR for 24 h. After harvest, cells were resuspended with the binding buffer containing 5 ng/µL of annexin-V-fluorescein isothiocyanate (FITC) and 20 µg/mL of PI for 30 min incubation. Subsequently, 400 µL of PBS was added and resuspended for flow cytometer analysis (BD Accuri™ C6; Becton-Dickinson) and a BD Accuri™ C6 Software (version 1.0.264).

Apoptosis was monitored by western blotting in terms of cleaved PARP expression, which was a apoptosis marker [20]. Western blotting was performed as described previously [35]. Western blotting was performed as described previously [34]. Briefly, the 20 µg protein lysates were separated by 10% SDS-polyacrylamide gel electrophoresis. After electrotransferring, PVDF membranes were probed with monoclonal antibody specific for uncut/cleaved forms of the poly ADP-ribose polymerase (PARP) (1:1000; #9542, Cell Signaling Technology; Beverly, MA, USA) and the cleaved PARP (Asp214) (D64E10) XP^®^ Rabbit mAb (1:1000; #5625, Cell Signaling Technology), glyceraldehyde-3-phosphate dehydrogenase (GAPDH) (1:10,000; GeneTex inc.; San Antonio, TX, USA). Signals were detected by the Advansta WesternBright™ ECL Western blotting detection kit (Menlo Park, CA, USA).

### 3.5. Intracellular ROS Level 

ROS detection was based on the fluorescence change of dye as described [14]. After seeding overnight, cells were incubated with or without MESR for 6 h or 12 h. After washing, cells were incubated with 100 nM of 2′,7′-dichlorodihydrofluorescein diacetate (DCFH-DA) in the culture incubator for 30 min. After harvest and washing, cells were resuspended in PBS for flow cytometer analysis (BD Accuri™ C6).

### 3.6. Mitochondrial Membrane Potential (MMP)

MMP was analyzed as described [39]. After seeding overnight, cells were incubated with MESR. Cells were incubated with A) in an incubator for 20 min. After washing, cells were resuspended in PBS for flow cytometer analysis (BD 50 nM of DiOC_2_ (3) provided in the MitoProbe™ DiOC_2_ (3) assay kit (Invitrogen, Eugene, OR, US Accuri™ C6).

### 3.7. Statistical Analysis

All statistics were based on the comparison between the drug-treated data with the controls using Student’s *t*-test.

## 4. Conclusions

MESR was proved to be antiproliferative against oral cancer Ca9-22 cells coupled with apoptosis, ROS overexpression, and mitochondrial dysfunction effects. MESR thus has potential to be a supplement for oral cancer therapy.

## References

[1] Yen C.Y., Chen C.H., Chang C.H., Tseng H.F., Liu S.Y., Chuang L.Y., Wen C.H., Chang H.W. (2009). Matrix metalloproteinases (MMP) 1 and MMP10 but not MMP12 are potential oral cancer markers. Biomarkers.

[2] Yen C.Y., Huang C.Y., Hou M.F., Yang Y.H., Chang C.H., Huang H.W., Chen C.H., Chang H.W. (2013). Evaluating the performance of fibronectin 1 (FN1), integrin alpha4beta1 (ITGA4), syndecan-2 (SDC2), and glycoprotein CD44 as the potential biomarkers of oral squamous cell carcinoma (OSCC). Biomarkers.

[3] Okui T., Shimo T., Fukazawa T., Hassan N.M., Honami T., Ibaragi S., Takaoka M., Naomoto Y., Sasaki A. (2012). Novel HSP90 inhibitor NVP-AUY922 enhances the anti-tumor effect of temsirolimus against oral squamous cell carcinoma. Curr. Cancer Drug Targets.

[4] Hu Y., McIntosh G.H., le Leu R.K., Nyskohus L.S., Woodman R.J., Young G.P. (2013). Combination of selenium and green tea improves the efficacy of chemoprevention in a rat colorectal cancer model by modulating genetic and epigenetic biomarkers. PLoS One.

[5] Lee J.C., Hou M.F., Huang H.W., Chang F.R., Yeh C.C., Tang J.Y., Chang H.W. (2013). Marine algal natural products with anti-oxidative, anti-inflammatory, and anti-cancer properties. Cancer Cell Int..

[6] Park H.Y., Lim C.W., Kim Y.K., Yoon H.D., Lee K.J. (2006). Immunostimulating and anticancer activities of hot water extract from *Capsosiphon fulvescens*. J. Kor. Soc. Appl. Biol. Chem..

[7] Costa L.S., Fidelis G.P., Telles C.B., Dantas-Santos N., Camara R.B., Cordeiro S.L., Costa M.S., Almeida-Lima J., Melo-Silveira R.F., Oliveira R.M. (2011). Antioxidant and antiproliferative activities of heterofucans from the seaweed *Sargassum filipendula*. Mar. Drugs.

[8] Khan Z., Bhadouria P., Bisen P.S. (2005). Nutritional and therapeutic potential of Spirulina. Curr. Pharm. Biotechnol..

[9] Zandi K., Ahmadzadeh S., Tajbakhsh S., Rastian Z., Yousefi F., Farshadpour F., Sartavi K. (2010). Anticancer activity of *Sargassum oligocystum* water extract against human cancer cell lines. Eur. Rev. Med. Pharmacol. Sci..

[10] Renju G.L., Muraleedhara Kurup G., Bandugula V.R. (2014). Effect of lycopene isolated from *Chlorella marina* on proliferation and apoptosis in human prostate cancer cell line PC-3. Tumour Biol..

[11] Namvar F., Mohamad R., Baharara J., Zafar-Balanejad S., Fargahi F., Rahman H.S. (2013). Antioxidant, antiproliferative, and antiangiogenesis effects of polyphenol-rich seaweed (*Sargassum muticum*). Biomed. Res. Int..

[12] Konickova R., Vankova K., Vanikova J., Vanova K., Muchova L., Subhanova I., Zadinova M., Zelenka J., Dvorak A., Kolar M. (2014). Anti-cancer effects of blue-green alga *Spirulina platensis*, a natural source of bilirubin-like tetrapyrrolic compounds. Ann. Hepatol..

[13] Yeh C.C., Tseng C.N., Yang J.I., Huang H.W., Fang Y., Tang J.Y., Chang F.R., Chang H.W. (2012). Antiproliferation and induction of apoptosis in Ca9-22 oral cancer cells by ethanolic extract of *Gracilaria tenuistipitata*. Molecules.

[14] Yeh C.C., Yang J.I., Lee J.C., Tseng C.N., Chan Y.C., Hseu Y.C., Tang J.Y., Chuang L.Y., Huang H.W., Chang F.R. (2012). Anti-proliferative effect of methanolic extract of *Gracilaria tenuistipitata* on oral cancer cells involves apoptosis, DNA damage, and oxidative stress. BMC Complement. Altern. Med..

[15] Kim J.Y., Yoon M.Y., Cha M.R., Hwang J.H., Park E., Choi S.U., Park H.R., Hwang Y.I. (2007). Methanolic extracts of *Plocamium telfairiae* induce cytotoxicity and caspase-dependent apoptosis in HT-29 human colon carcinoma cells. J. Med. Food.

[16] Ara J., Sultana V., Qasim R., Ahmad V.U. (2002). Hypolipidaemic activity of seaweed from Karachi coast. Phytother. Res..

[17] Sayers E.W., Barrett T., Benson D.A., Bryant S.H., Canese K., Chetvernin V., Church D.M., DiCuccio M., Edgar R., Federhen S. (2009). Database resources of the National Center for Biotechnology Information. Nucleic Acids Res..

[18] Kamenarska Z., Taniguchi T., Ohsawa N., Hiraoka M., Itoh N. (2007). A vanadium-dependent bromoperoxidase in the marine red alga *Kappaphycus alvarezii* (Doty) Doty displays clear substrate specificity. Phytochemistry.

[19] Pooja S. (2014). Algae used as medicine and food-a short review. J. Pharm. Sci. Res..

[20] O’Brien M.A., Moravec R.A., Riss T.L. (2001). Poly (ADP-ribose) polymerase cleavage monitored *in situ* in apoptotic cells. Biotechniques.

[21] Zakaria N.A., Ibrahim D., Sulaiman S.F., Supardy N.A. (2011). Assessment of antioxidant activity, total phenol content and *in vitro* toxicity of Malaysian red seaweed. J. Chem. Pharm. Res..

[22] Wang T., Jónsdóttir R., Ólafsdóttir G. (2009). Total phenolic compounds, radical scavenging and metal chelation of extract from Icelandic seaweed. Food Chem..

[23] Khan N., Afaq F., Mukhtar H. (2008). Cancer chemoprevention through dietary antioxidants: Progress and promise. Antioxid. Redox Signal..

[24] Yang J.I., Yeh C.C., Lee J.C., Yi S.C., Huang H.W., Tseng C.N., Chang H.W. (2012). Aqueous extracts of the edible *Gracilaria tenuistipitata* are protective against H_2_O_2_-induced DNA damage, growth inhibition, and cell cycle arrest. Molecules.

[25] Lu L.Y., Ou N., Lu Q.B. (2013). Antioxidant induces DNA damage, cell death and mutagenicity in human lung and skin normal cells. Sci. Rep..

[26] Fox J.T., Sakamuru S., Huang R., Teneva N., Simmons S.O., Xia M., Tice R.R., Austin C.P., Myung K. (2012). High-throughput genotoxicity assay identifies antioxidants as inducers of DNA damage response and cell death. Proc. Natl. Acad. Sci. USA.

[27] Dellai A., Deghrigue M., Bouraoui A. (2013). Evaluation of the antiproliferative activity of methanol extract and its fractions from the mediterranean seaweed, *Halurus equisetifolius*. Int. J. Pharm. Pharm. Sci..

[28] Yang Y., Zhang Z., Li S., Ye X., Li X., He K. (2014). Synergy effects of herb extracts: Pharmacokinetics and pharmacodynamic basis. Fitoterapia.

[29] Wagner H. (2011). Synergy research: Approaching a new generation of phytopharmaceuticals. Fitoterapia.

[30] Gorrini C., Harris I.S., Mak T.W. (2013). Modulation of oxidative stress as an anticancer strategy. Nat. Rev. Drug Discov..

[31] Trachootham D., Alexandre J., Huang P. (2009). Targeting cancer cells by ROS-mediated mechanisms: A radical therapeutic approach?. Nat. Rev. Drug Discov..

[32] Deavall D.G., Martin E.A., Horner J.M., Roberts R. (2012). Drug-induced oxidative stress and toxicity. J. Toxicol..

[33] Kurt O., Ozdal-Kurt F., Tuglu M., Akcora C. (2014). The cytotoxic, neurotoxic, apoptotic and antiproliferative activities of extracts of some marine algae on the MCF-7 cell line. Biotech. Histochem.

[34] Kajstura M., Halicka H.D., Pryjma J., Darzynkiewicz Z. (2007). Discontinuous fragmentation of nuclear DNA during apoptosis revealed by discrete “sub-G1” peaks on DNA content histograms. Cytometry A.

[35] Chiu C.C., Haung J.W., Chang F.R., Huang K.J., Huang H.M., Huang H.W., Chou C.K., Wu Y.C., Chang H.W. (2013). Golden berry-derived 4beta-hydroxywithanolide E for selectively killing oral cancer cells by generating ROS, DNA damage, and apoptotic pathways. PLoS One.

[36] Chen B.H., Chang H.W., Huang H.M., Chong I.W., Chen J.S., Chen C.Y., Wang H.M. (2011). (−)-Anonaine induces DNA damage and inhibits growth and migration of human lung carcinoma h1299 cells. J. Agric. Food Chem..

[37] Chiu C.C., Liu P.L., Huang K.J., Wang H.M., Chang K.F., Chou C.K., Chang F.R., Chong I.W., Fang K., Chen J.S. (2011). Goniothalamin inhibits growth of human lung cancer cells through DNA damage, apoptosis, and reduced migration ability. J. Agric. Food Chem..

[38] Huang H.W., Chung Y.A., Chang H.S., Tang J.Y., Chen I.S., Chang H.W. (2014). Antiproliferative effects of methanolic extracts of *Cryptocarya concinna* Hance roots on oral cancer Ca9-22 and CAL 27 cell lines involving apoptosis, ROS induction, and mitochondrial depolarization. Sci. World J..

[39] Yen C.Y., Chiu C.C., Haung R.W., Yeh C.C., Huang K.J., Chang K.F., Hseu Y.C., Chang F.R., Chang H.W., Wu Y.C. (2012). Antiproliferative effects of goniothalamin on Ca9-22 oral cancer cells through apoptosis; DNA damage and ROS induction. Mutat. Res..

